# Gait physiotherapy with motor imagery in people with Parkinson’s disease: a protocol for randomized control GAITimagery trial

**DOI:** 10.3389/fneur.2024.1508043

**Published:** 2025-01-17

**Authors:** Constanza San Martín Valenzuela, Esperanza Ramírez Murcia, Estela Aznar-Requena, Dalia García Sotolongo, Rebeca Rosas-Martín, M. Luz Sánchez-Sánchez

**Affiliations:** ^1^Department of Physiotherapy, Faculty of Physiotherapy, University of Valencia, Valencia, Spain; ^2^Unit of Personal Autonomy, Dependency and Mental Disorder Assessment, Faculty of Medicine, University of Valencia, INCLIVA Biomedical Research Institute, Valencia, Spain; ^3^Centro Investigación Biomédica en Red de Salud Mental, CIBERSAM, Madrid, Spain; ^4^Asociación Parkinson Valencia Neurorehabilitation Center, Valencia, Spain; ^5^Physiotherapy in Motion, Multispeciality Research Group (PTinMOTION), Department of Physiotherapy, University of Valencia, Valencia, Spain

**Keywords:** Parkinson’s disease, gait, motor imagery, physiotherapy, physical therapy, action observation, quality of life, biomechanic

## Abstract

**Introduction:**

According to people with Parkinson’s disease (PD), gait impairments are the most disabling motor symptoms of PD. Recently, motor imagery (MI) has gained notoriety as a gait training technique due to the flexibility of its use, however, it has not been demonstrated that causes a superior effect when included in physiotherapy. This study aims to determine if gait training combined with MI has a greater effect on the gait of people with PD than just gait training.

**Methods:**

The GAITimagery is designed as a double-blind, randomized control trial, including a convenience sample in 2 parallel groups (1:1) with two interventions of 2 sessions per week during 6-week and 8-week follow-up. The initial recruitment will be 88 participants with idiopathic PD and unimpaired cognition state, who will be randomly divided into two groups: GAITimagery (GiG) or the active control Gait group (GaG). Both will perform the same gait exercises but only GiG will include MI training. Gait speed is the primary outcome, while Maximum gait speed (m/s) and Gait speed variability are the secondary results. The tertiary outcomes are related to Quality of life, Daily life activities, Freezing of gait, Balance, Mobility, and Gait performance measures to psychometrics and biomechanics instruments. All results will be measured at baseline (t0), post-training (t1), and follow-up assessment (t2) 8 weeks after finished physiotherapy programs.

**Discussion:**

The GAITimagery program standardizes the application of MI exercises related to the improvement of parkinsonian gait at the same time that monitoring the vividness referred by the participants session by session. The effectiveness of this MI-exclusive program includes subjective and objective measurement tools to detect minimal changes after training. This still-to-be-finish study will support the therapeutic decisions on whether or not to allocate session time to imagery exercises depending on the effect size achieved and the comparison with a control gait training.

## 1 Introduction

Parkinson’s disease (PD) is a neurodegenerative, chronic, progressive, and multisystemic disease, with motor and non-motor signs, which affects functional mobility and eventually causes disability and dependence (1, 2). Although the main therapeutic strategy is pharmacological, the effectiveness of oral Levodopa is reduced as the disease progresses (3) and the long-term use has secondary effects (4). On the other hand, physiotherapy is decisive in extending the time of functionality and independence (5), and avoiding major complications such as falls, hospitalizations, and greater overall deterioration (6). Accordingly, patients report gait impairments as the most disabling motor symptoms of the disease (7), which can appear from early onset onwards (8), thus, new gait physiotherapy strategies are continually under study. Recently, motor imagery (MI) has gained notoriety due to the flexibility of its use. MI is the mental rehearsal of action in the absence of overt motor output (9, 10), which may be differentiated into visual motor imagery (VMI) and kinesthetic motor imagery (KMI). VMI relates to the generation of visual representations of motion, while KMI relates to the sensations associated (11). MI is based on activating similar cortical networks as motor execution (11, 12), which would allow people with motor disorders to facilitate the practice of normal movement patterns through MI, as in advanced stages of PD. This premise implies the education of corrected movement along the MI training, to perform the mental practice with a non-altered execution. Related to this, Action Observation (AO) usually is part of MI programs, since observing a scene in motion without moving support learning and accuracy of MI (13).

Previous authors have studied through randomized controlled trials, the effectiveness of MI interventions in PD combined with gait exercises or regular physical therapy (14–17), dual-tasks (18–20), virtual reality (21, 22), and neurofeedback (23, 24). From those works that compare gait training or conventional physical practice with MI versus without MI, they report no differences post-training between groups in motor function after 24 sessions (17), 12 sessions (14), 6 sessions (16), and a single training session (15). Although these designs would allow it to analyze the proportion of improvements attributed to the MI technique, there are methodological considerations that may condition the previously mentioned results. The first of them, is the PD severity presented by the participants in the informed works, which ranges from mild to moderate. Although gait alterations can be observed from the initial stages of PD (8, 25, 26), these may be insufficient to show significant differences between groups post-training. Regarding to this, the non-instrumental measurement tools used (14, 16, 17) may be inadequate to detect small changes when participants do not present severe gait impairments. On the other hand, authors who include MI in gait physiotherapy and use biomechanics assessment tools, only report the effects of a single therapy session (15), therefore a minimum training dose is not given (27) that allows differences to be observed.

Another reason that may contribute to the fact that no differences are observed between therapy with and without MI is the non-stratified randomization of the participants (14–17), since decompensated groups could mask a possible superior effect of the gait physiotherapy supported by MI. Finally, and related to the sample size analyzed in the literature, these do not exceed 50 subjects (14–17), hence the sample size may underestimate the number of participants necessary to observe differences between groups if this is based on intragroup post-intervention change (14). Given the above, this study aims to determine the effect of adding MI exercises to gait training of people with mild-to-severe PD. We hypothesize that MI added to gait rehabilitation achieves a greater effect than isolated gait training in people with PD, and that this effect increases as the disease progresses, even when AO and walking exercise are exactly the same in control and experimental group.

## 2 Methods

### 2.1 Study design

**Figure 1:**
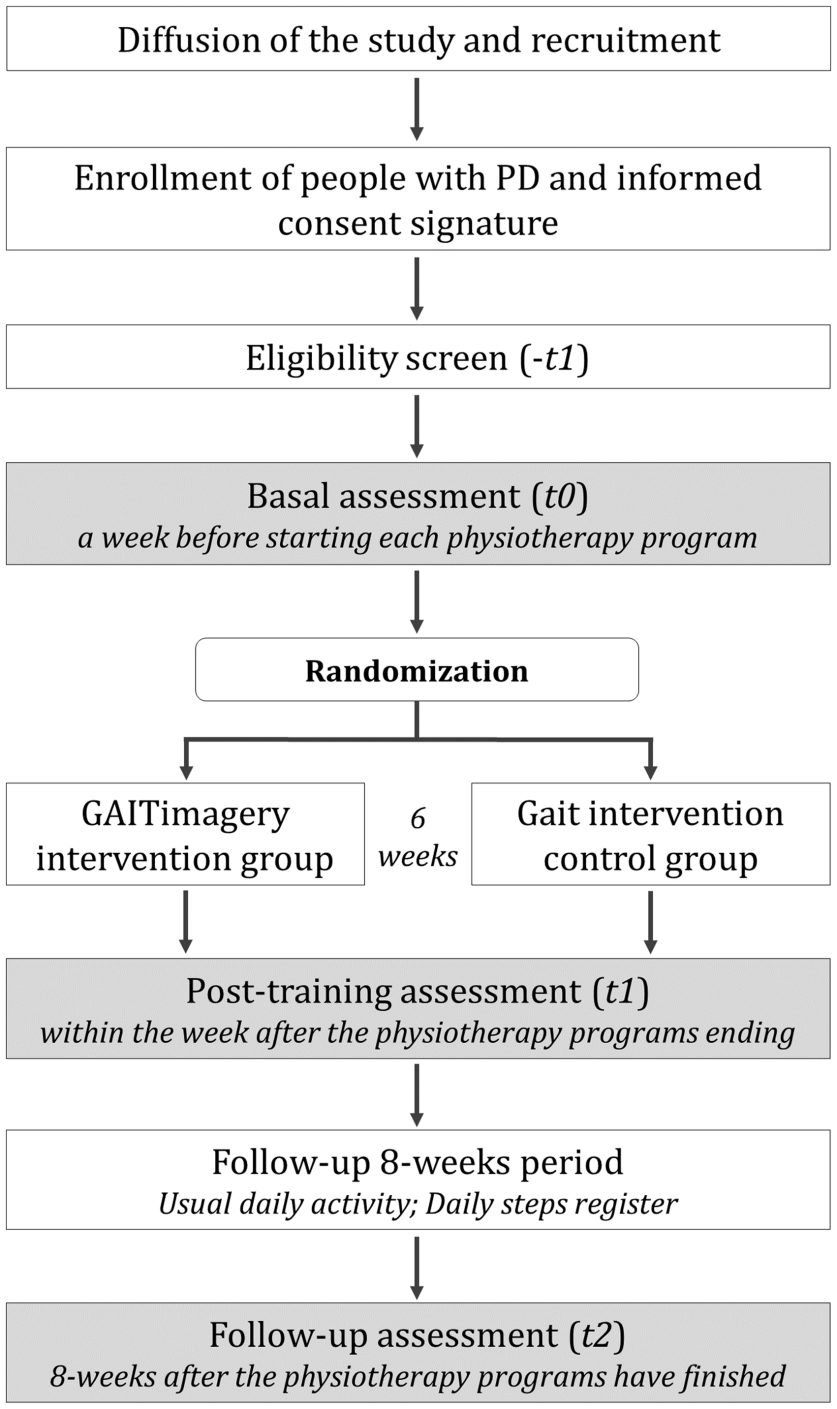
Trial design and participants flow. Flow of the study intervention and assessment.

The GAITimagery is designed as a double-blind, randomized control trial with 6 weeks of intervention (two sessions weekly) and 8 weeks of follow-up. The study considered a convenience sample (specifically, modal instance sampling) in two parallel groups (1:1), and an additional matched-healthy group (Figure 1). This protocol used the Standard Protocol Items for Randomized Trials (SPIRIT) guidelines (28) (Supplementary material 1) and the Consolidated Standards of Reporting Trials statement for randomized controlled trials (29) as a guide to be designed. The Human Research Ethics Committee of the Experimental Research Ethics Commission of the University of Valencia approved all the procedures with the number 1557673 (Supplementary material 2) on 11 February 2021. Then it was registered on ClinicalTrials.gov with the number NCT04788693.

This study will be started with a baseline assessment session (t0) followed by random allocation to one of two study groups: the GAITimagery group (GiG) or the active control Gait group (GaG). The first one will perform 6-weeks of gait physiotherapy combined with MI. The second one will perform the same gait exercise of GiG without the MI techniques. At the end of the interventions, a post-training evaluation (t1) will be carried out, and subsequently, a follow-up assessment (t2) 8-weeks after the physiotherapy programs have finished. Additionally, a third group of healthy matched-sex and age participants will be assessed to compare the performance of PD participants.

### 2.2 Participants, interventions, and outcomes

#### 2.2.1 Setting and eligibility criteria

Participants will be recruited from the local hospitals and rehabilitation centers of the city of Valencia. The eligibility criteria assessment, obtaining informed consent (Supplementary material 3), assessments, and intervention of both groups will be carried out in the facilities of the Physiotherapy Department at the University of Valencia. The recruitment and eligibility criteria assessments will be accomplished by a neurologist from the hospitals who regularly carry out pharmacological and disease supervision. The inclusion criteria are: (1) diagnosed with PD according to the United Kingdom Parkinson’s Disease Society Brain Bank diagnostic scale; (2) independent walk in a 10-meter corridor; (3) normal cognitive state, determined by the Mini-Mental State Examination with a score ≥ 25 and the Montreal Cognitive Assessment ≥26; and (4) stable medication from the month prior to the start of the study until the t2 assessment. Likewise, the exclusion criteria are: (1) additional neurological condition different from PD; (2) disease or musculoskeletal acute alteration that limits mobility or balance; (3) lower extremities asymmetries >1 cm; (4) report pain on the Visual Analog Scale; (5) suffer from blindness, deafness or any other visual/hearing impairment or pathology that may influence the ability to understand instructions and carry them out; (6) significant tremor that may interrupt the MI exercise; and (7) to perform other physical therapies or sports during the trial or in the 2 months before.

We expect patient recruitment to begin in September 2025 and completed in July 2026. All patients are assumed to have completed baseline testing in September 2026. In addition, the recruitment of healthy people counterparts will be carried out once the participation of patients with PD ends, to recruit people with similar characteristics of age, weight, height, and gender. This procedure will be carried out at the local city’s Municipal Senior Activity Center. The inclusion and exclusion criteria are the same for patients, except those related to the PD.

#### 2.2.2 Intervention

Two physiotherapy programs, GAITimagery and gait physiotherapy program, have been scheduled to take place over 6-week period. Each program involves two non-consecutive sessions per week, lasting 60 min each, which makes a total of 12 sessions. Additionally, this study proposes an 8-week follow-up period, which extends beyond the intervention phase. The rationale for this design is based on evidence that the benefits of gait exercises for PD emerge rapidly and are sustained over time, achieving short-term improvements in 12 to 14 training sessions over 2 to 4 weeks (27), which can persist for 3 to 12 months after treatment completion (30). The GAITimagery program will be performed for the GiG, while the GaG will develop the gait physiotherapy program without the MI exercises. To replicate the same conditions in both programs, the sessions will be done in groups of two people with the same PD severity according to the Hoehn & Yahr (H&Y) scale. Each program will be developed by different physiotherapists with an assistant, and supervised by a third physical therapist who checks that the walking exercises applied in both groups are the same. The sessions of both programs also have the same structure: warm-up (10′), central phase (45′), and cool-down (5′). Both interventions will be performed in a quiet environment, in a 7x13m room located in an area isolated from outside noise to promote the concentration of participants during the sessions. The warm-up and cool-down are the same in both programs. The essential aspects of physiotherapy in PD will be included in the warm-up section, related to respiratory exercises, dissociation of the shoulder and pelvic girdle, transfers and postural changes, joint mobility, balance, and strengthening (31). The cool-down part cover self-assisted stretching.

**Figure 2:**
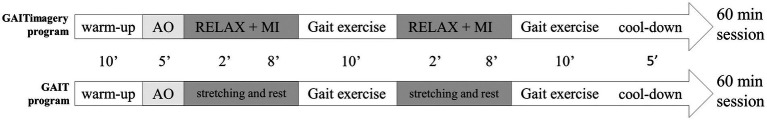
Organization of the GAITimagery and control gait program session. Both programs have the same structure with the exception of the Relaxation and Motor imagery section, which will be only performed in the GAITimagery program. AO, action observation. MI, motor imagery.

The central phase includes the gait training itself, in which the first 5′ will be dedicated to the explanation the session objectives and to applying the observation part of AO technique through previously prepared videos of Parkinsonian and normal gait. The purpose of AO in our protocol is to prepare participants who will subsequently perform MI exercises. However, as AO may have an effect on the physical performance of gait exercises by itself (32), both groups will perform the observation part at the beginning of gait training (Figure 2). Within the stipulated 5′, a laptop will be used to show a video focusing on the gait characteristic to be worked on in the session (whether spatiotemporal or kinematic), performed by a subject without pathologies. The physiotherapist will support the visual information with a theoretical explanation of the video content. Next, a video of the same characteristic will be shown but performed by a person with PD. The physiotherapist will explain to participants the features to be corrected. Finally, three additional videos will be shown where participants must say whether it corresponds to a normal or altered pattern. The active part of the AO will be carried out through the gait exercises proposed for each session. The walking exercises for both groups are related to the spatiotemporal and cinematic impairment of the Parkinsonian gait (31), hence are the following (33, 34): (1) step length increase through visual cues and circuit with obstacles of different sizes; (2) cadence control with digital metronome; (3) speed training over 1.00 m/s controlling the stride length and cadence; (4) posture correction during walking; (5) arm swing and shoulder girdle dissociation with proprioceptive and auditory cueing; (6) promotion the lower limb kinematic milestones of stance and swing phase, and (7) Dynamic balance during gait. All the gait exercises will be adapted to the severity of PD.

**Figure 3:**
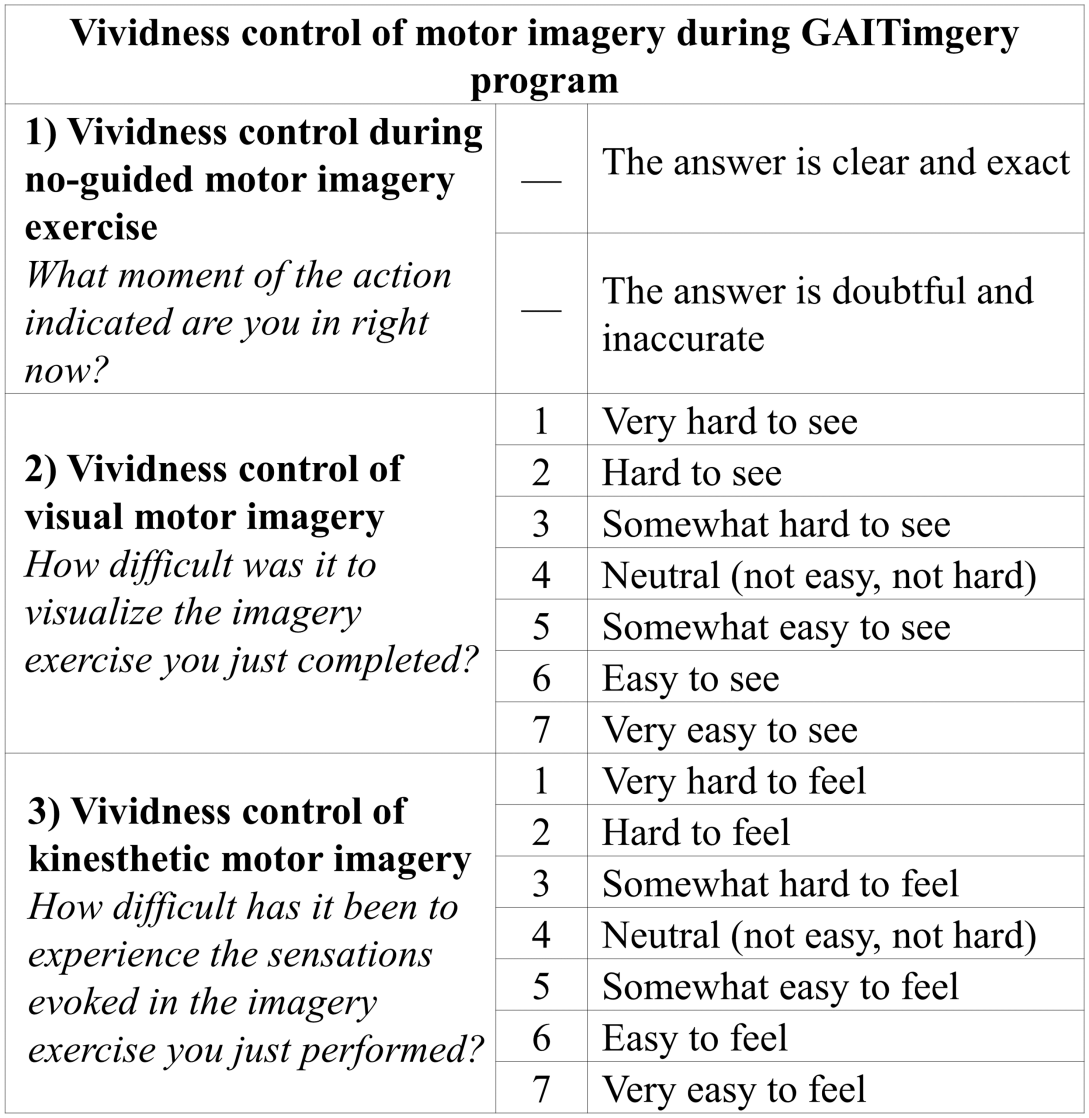
Vividness control of motor imagery exercises. The items aims to control whether the GAITimagery program participants are being able to carry out the motor imagery exercises effectively.

Additionally, the GAITimagery program consists of interspersing the physical exercise of walking with periods of MI in each session. Before MI practice, a relaxation exercise will carried out in supine position on a stretcher, obtaining in each session two complete cycles of 2’ of relaxation, 8′ of MI, and 10′ of physical walking exercise related to the treatment objective that is reinforced in the MI period (Figure 2). The MI exercises will consist of imaging a normal gait sequence focused on visualizing a requested movement (VMI) alternating with the focus on the sensation and muscle contraction associated with performing the movement indicated (KMI). MI and relaxation exercises will be guided by the voice of the physiotherapist, who will indicate what movement to imagine and the execution that should be evoked, based on the correction of the gait parameters previously studied in the AO period. All the instructions will be standardized and the physiotherapist will read it from a script. When the goal session is kinematic aspects of gait, the physiotherapist will simply describe to the participants the movement they should imagine, helping them to create a mental image with sufficient detail. When the session’s goal involves speed training, the vocal guidance will include a cadence of the steps, set by a digital metronome that the physiotherapist will listen to through headphones. Between the 8′ of MI guided by the physiotherapist, 30-s spaces will be given without vocal guidance so that participants can silently visualize the required exercise. In this no-guided MI interval, the imagery activity will be monitored through a question about the performance (35), to check the precision of MI (“What moment of the action indicated are you in right now?”). Additionally, at the end of each MI block, the participant will give feedback through two Likert-type questions (36, 37) related to the MI exercise performed (“How difficult was it to visualize the imagery exercise you just completed?” and “How difficult has it been to experience the sensations evoked in the imagery exercise you just performed?”) (Figure 3).

Finally, in each physiotherapy session, the time elapsed since the last intake of antiparkinsonian medication for each GiG and GaG participant will be recorded. Likewise, the number of sessions completed during the program will be noted for each person.

#### 2.2.3 Outcomes and participant timeline

The participants will be evaluated in ON-medication state (1 h after the dopaminergic dose) on the schedule described in Figure 1. Two physiotherapists will perform the assessments, which are different from the treating therapists, and blinded to the group assignment. After signing informed consents, a neurological clinical interview for PD (38) will be performed during the eligibility screening (−t1), in addition to the register of the anthropometric data lower-limbs length (during standing position from the anterior superior iliac spine to the medial malleolus), weight, and height. For this purpose, a wall stadiometer and a TANITA SC-240MA scale will be used. Also, at -t1, the severity of the disease will be evaluated with Hoehn & Yahr (39), its modified version (40), and the Movement Disorders Society Unified Parkinson’s Disease Rating Scale (41), as well as the cognitive status through the Montreal Cognitive Assessment (42, 43) and the MiniMental Parkinson Test (44).

**Figure 4:**
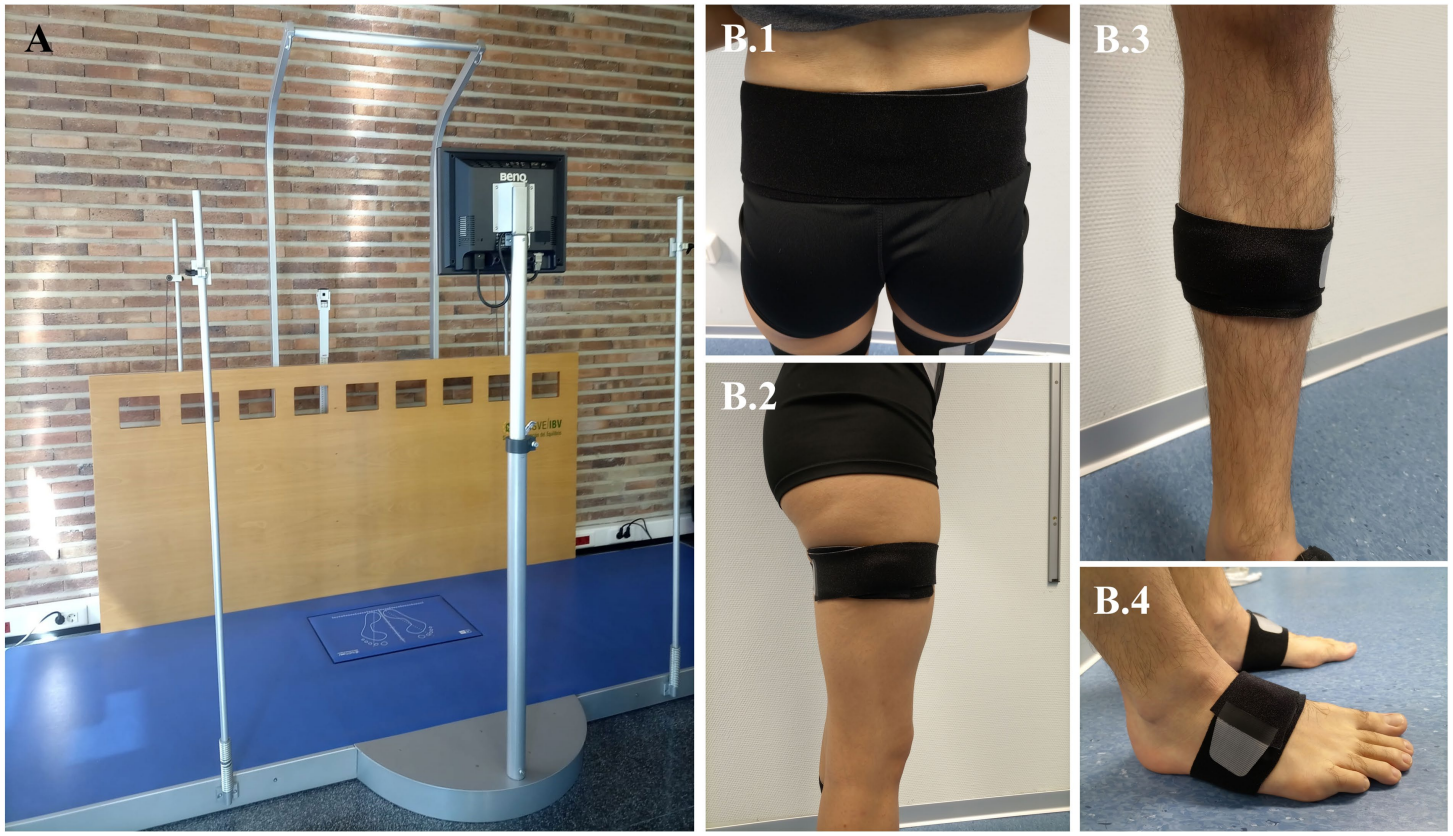
Biomechanical model and anatomical placement of motion capture markers. **(A)** Dynamometric platform used in the biomechanical gait assessment. **(B)** Instrumentation of Inertial Sensor Units measure, which will be attached by elastic straps provided by the manufacturer. Each sensor must be placed at the following zones: **(B.1)** Lumbar, located just below the spinosa of the lumbar vertebra 5. **(B.2)** Thighs, located at the lower limit of the upper third of the thigh, on its lateral surface. It is considered as the total measurement of the thigh from the greater trochanter to the interarticular line. **(B.3)** Legs, placed at the midpoint of the leg measured from the interarticular line to the medial malleolus. The sensor is positioned just medial to the tibial border. **(B.4)** Feet, positioned on the dorsum of the foot, four centimeters from the intersection of the inter-malleolar line, above a line projected toward the fourth toe.

During baseline (t0), post-6-week-training (t1), and follow-up (t2) will be measured (Table 1): (1) Perception of health-related quality of life due to PD using the Parkinson disease questionnaire (45, 46); (2) Activities in daily life employing the Schwab & England scale to indicate the percentage of functional independence in the performance of basic and instrumental activities of daily living (47, 48); (3) Freezing of gait by means of the Freezing of gait questionnaire (49); (4) Clinical performance of gait evaluated using the Tinetti mobility test and the Dynamic Parkinson gait scale (50, 51); (5) Dynamic balance assessed with the Tinetti mobility test and the MiniBEST test (50, 52); (6) Functional mobility evaluated with Timed up and go test (53); and (7) Biomechanical gait pattern measured with instrumental techniques to record spatiotemporal, kinematic, and kinetic variables (Table 1). In this respect, to record the ground reaction forces and gait speed during walking, it will be used a dynamometric platform (Dinascan/IBV Biomechanics Institute of Valencia, Valencia, Spain) and the NedAMH®/IBV software (version 5.5.0, Biomechanics Institute of Valencia, Valencia, Spain), which uses two red-light photocells too. The dynamometric platform (Figure 4A) is located in a 8 meter-walkway, in which the participants will have to walk barefoot (socks only) at self-select comfortable speed until completing at least 10 repetitions, to obtain 5 registers with each footprint. On the other hand, to record kinematics parameters of gait (Table 1), 7 Magneto-inertial measurement units (IMU) XSENS DOTs (XENS, Enschede, The Netherlands) will be employed along with their application for mobile phones to manage the measurement record (Movella DOT, version 2023.6.1). Each IMU includes a ± 2000°/s 3-axis-gyroscope, ±16 g 3-axis-accelerometer, ±8 Gauss 3-axis-magnetometer, has a dimension of 36.3 × 30.35 × 10.8 mm, and weighs 11.2 g. The gait assessment with IMUs will be carried out at the same time as the dynamometric measure. The IMUs set will be placed on the lumbar zone, thighs, legs, and feet segments, as Figure 4B shows, attached by elastic straps provided by the manufacturer to prevent them from slipping. The instruction to initiate the gait register will be given to the participants 3 seconds after starting the registration with the mobile app, through the command “start walking.” At the end of the walkway, participants will be instructed to remain standing for three other seconds, after which the recording with the IMUs will be stopped. All raw data from IMUs will be processed with Python (Python Software Foundation, version 3.7.17, 2023) to obtain the outcomes in Table 1.

**Table 1:** Summary of outcomes, measurement instrument, and assessment times.

Domain / Outcomes	Measurement instrument	Study period			
		-t1	t0	t1	t2
Informed consents		x			
Anamnesis					
Personal information	Clinical interview for Parkinson’s disease	x			
Habits and working life		x			
Disease start and initial sign		x			
Parkinson’s disease severity	H&Y; modified H&Y; MDS-UPDRS-III	x			
General cognitive state	MMP; MoCA	x			
Eligibility screen	Participant criteria	x			
Anthropometric data (weigh, height, and lower limb length)	Wall stadiometer, TANITA SC-240MA scale, and tape measure	x			
Quality of life					
Activities of daily life	Schwab & England scale		x	x	x
Perception of QoL due to PD	PDQ-39		x	x	x
Gait clinical performance					
Freezing of gait	FOG-Q		x	x	x
Gait	TMT gait; DYPAG		x	x	x
Dynamic balance	TMT balance; MiniBEST test		x	x	x
Functional mobility	TUG		x	x	x
Gait biomechanical performance					
Speed (m/s) _Primary outcome_	Dynamometric platform		x	x	x
Speed variability _Secondary outcome_					
Maximum gait speed (m/s) _Secondary outcome_			x	x	x
Stance time (s)			x	x	x
Weight-acceptance GRF (*N*; weight%)			x	x	x
Midstance GRF (*N*; weight%)			x	x	x
Push-off GRF (*N*; weight%)			x	x	x
Breaking GRF (*N*; weight%)			x	x	x
Propulsion GRF (*N*; weight%)			x	x	x
Cadence (steps/min)	Magneto-inertial measurement units		x	x	x
Stride and step length (m)			x	x	x
Stance and Swing phase (s; gait cycle %)			x	x	x
Double support time (s; gait cycle %)			x	x	x
Range of motion of lower limb joint (°)			x	x	x
Maximum ankle dorsiflexion during swing (°)			x	x	x
Maximum knee flexion during swing (°)			x	x	x
Maximum hip extension during stance (°)			x	x	x
Maximum hip flexion during swing (°)			x	x	x

H&Y, Hoehn y Yahr scale; MDS-UPDRS-III, Movement Disorders Society Unified Parkinson’s Disease Rating Scale, part three; MMP, Minimental Parkinson test; MoCA, Montreal Cognitive Assessment; PDQ-39, Parkinson disease questionnaire; FOG-Q, Freezing of gait questionnaire; TMT, Tinetti mobility test; DYPAG, Dynamic Parkinson gait scale; TUG, Timed up and go test; GRF, Ground reaction force.

The study’s primary outcome is Gait speed at the post-training time (primary time point), defined as the distance traveled by the body per unit of time (m/s). The Gait speed is an objective variable, easy to measure with or without instrumental equipment, and considered a relevant indicator of functional and health status (54, 55). In addition, the secondary outcomes of this trial are the Maximum gait speed (m/s) (56) and the Gait speed variability, calculated through the coefficient of variation ([standard deviation/mean] × 100) (57), which indicates how stable or repeatable the participants are when walking. These variables could be more sensible to gait impairments (58) and changes after treatment (57). On the other hand, the tertiary outcomes are those from the spatiotemporal, kinematics, and kinetics gait analysis, in addition to the scores of the scales and questionnaires proposed in Table 1.

#### 2.2.4 Sample size and recruitment

Sample size calculation has been done with the G*Power software (Universität Kiel, Germany, Version 3.1) (59), based on the expected differences in gait performance between experimental and control groups. Due to the absence of statistical differences between groups in the previous works that compare gait physiotherapy with and without MI, the estimation was settled with a small expected size effect of *F* = 0.15 (60). A power of 80% and a type I statistical error of 5% were considered in a mixed factorial design (2 groups and 3 times assessments), which resulted in 74 participants as the total sample size. Considering 20% of drop-outs, the recruitment will be 44 people per group. For the participant’s recruitment, the research coordinator of the study will establish communication with local health centers to provide information and contact details about the GAITimagery study. The centers will be asked to distribute this information among potential participants through brochures, after which those interested in participating will have to communicate their intention by telephone to the coordinator research. After this, an appointment to evaluate the participation criteria and the severity of the disease will be given to the people who have established communication with the study. The recruitment process will remain open until the necessary number of people is met according to the sample size calculation, as long as it does not exceed 2 years. If this time ends, the preliminary results will be reported, informing the power of the study fulfilled with the sample obtained.

### 2.3 Assignment of intervention and blinding

A stratified randomization process will be performed by the external research, considering the H&Y stages. Because patient recruitment will occur over a prolonged period, to control the stratification, the randomization will be performed after 12 participants group recruitment, and this process will be repeated six times, with the exception that the last group of recruitment will be of 14 people instead of 12. The allocation in each group of recruitment will be 1:1, using a function of Microsoft Excel (V18.0, 2021. Excel; Microsoft Corp). To ensure masking, the raters physiotherapist, and data analysis researcher, will be blinded to the participant’s allocation. Although participants and treating physiotherapists cannot be totally blinded to the intervention performed, the hypothesis and objectives of the study will be hidden from them. At the same time, all participants will be instructed not to disclose information regarding their intervention to the raters’ physiotherapists. To control expectancy effects, it will be explained to patients that it is not yet determined which therapy is more effective. On the other hand, a consecutive number code will be assigned to the participants without disclosing the treatment group to which they belong.

### 2.4 Data collection, management data, and statistical analysis

An electronic template was prepared with the items from the clinical interview and results from scales and questionnaires, to ensure no missing data. All the assessments will be registered under a code assigned to each participant (coding). In this way, the data dump to a database is carried out under the supervision of one of the evaluators who, in turn, has the task of guaranteeing anonymization of the participants in the outcomes registry. Personal participants’ data from the clinical interview will be located separately from the main dataset on a local computer to protect confidentiality during all trial phases. The raw dataset will be maintained for 10 years after the completion of the trial with indefinite restricted access due to sensitive data. No individual data from participants will be openly available. Instead, all additional information to the results presented in future publications will be provided through the corresponding supplementary material to each publication. Lastly, the participants do not receive any incentives or compensation for participation in this study.

Statistical analyses will be performed using SPSS v.24 (SPSS Inc., Chicago, IL, USA). If adherence issues occur, all analyses will be evaluated using intention-to-treat principles, with a level of significance of 0.05. Mean and standard deviations (SD) will be calculated to inform all the continuous variables that have a normal distribution. On the contrary, continuous outcomes with an asymmetric distribution will be reported through the median. Outcomes from ground reaction forces will be standardized based on the weight of participants. Categorical results will be informed through frequency. To check for differences between groups on the demographic outcomes, multivariate one-way analysis with the between-subject factor *Group* will be conducted. Furthermore, to test for sex differences between groups, a chi-square test will be used.

A two-factor mixed Multivariate Analysis of Variance (MANOVA) will be conducted to analyze the factors effects on the outcomes registered at t0, t1, and t2. The within-subject factor is *Time* with three categories (Baseline, Postintervention and Follow-up). The between-subject factor is *Group* with two categories (GiG and GaG). When significant effects are found, Bonferroni will be used for post-hoc comparisons. Differences shall be declared statistically significant if *p* < 0.05. Before the referred statistical analysis above, the following assumptions will be checked: (1) Normality: the distributions of the residuals will be tested using the Shapiro–wilk test; (2) Sphericity: the homogeneity between variances of the differences between pairs of measures will be checked with the Mauchly sphericity test; (3) Homoscedasticity: homogeneity of variances will be evaluated using the Levene test; (4) Equality of covariance matrices: the equality of variance–covariance matrices across the cells formed by the between-subjects effects will be tested with the Box’s *M* test; (5) Absence of multicollinearity, the correlation level between dependent variables will be observed with the Pearson correlation test. If any of these assumptions are not met, the necessary statistical corrections will be used (61).

### 2.5 Monitoring

This study did not incorporate external professionals for data monitoring. Instead, data monitoring is carried out by an internal Data Monitoring Committee, integrated by (1) the research coordinator of the study, (2) the research co-coordinator, and (3) the data-analysis professional. Participant monitoring is carried out weekly by telephone on a non-therapy day, which allows us to promote participant retention and encourage participants to complete the follow-up. The following information is recorded: (1) unusual or new neurologic symptoms, (2) adverse events such as falls, (3) level of pain experimented during the week, (4) medical appointments and motive, (5) changes in medication; (6) level of physical activity outside of the study, (7) mood, anxiety, and depressive symptoms during the week and, (8) individual perception of the evolution process. The Data Monitoring Committee will decide whether trial participation should be discontinued, based on monitoring reports. If participants decide to withdraw, the Data Monitoring Committee will be in charge of collecting the reasons for abandonment.

### 2.6 Patient and public involvement

Patient and/or public were not involved in the design of this protocol.

## 3 Ethics and dissemination

All procedures were approved by the Ethics Committee of Research in Humans of the Ethics Commission in Experimental Research of University of Valencia on February 11, 2021 (Register number 1557673), in accordance with the principles of the World Medical Association’s Declaration of Helsinki, the Council of Europe Convention regarding human rights and the requirements established in Spanish legislation in the field of Biomedical research, personal data protection and bioethics. We did not consider future changes in the study protocol reported by the committee. Eligible participants are informed about the study and the privacy protection concept before participating. The physiotherapists in charge of assess the eligibility criteria will be responsible for obtaining the written informed consent (Supplementary material 3) from the participants before the first tests started. All the data and information collected regarding this trial are treated confidentially (blinded and encrypted) by the researchers connected to the study. Finally, all results from the trial will be published in international peer-reviewed scientific journals, regardless of whether these results are negative or inconclusive.

## 4 Discussion

This study protocol aims to determine the improvements in gait rehabilitation in people with PD, who integrate MI techniques into the treatment compared with a group gait training without MI. Functional neuroimaging has documented that MI and motor execution activate similar cortical networks as the primary motor cortex and pre-motor areas, including the supplementary motor area (11, 13, 62). Based on this, the MI could be especially useful in people who need to correct or reinforce movement patterns due to motor disorders, facilitating the right execution. Previous randomized control trials have studied the effect of gait physiotherapy with and without MI in their protocols, with no statistical differences between groups (14–17). This lack of differences that support the clinical decision to integrate MI into routine practice, may arise because of different methodological approaches. The first of them is the quality of the vividness that the participants have during the imagery exercise, conditioned by the inclusion of the AO phase. Since the patients must imagine a corrected movement pattern, education on the gait cycle is essential to ensure the integration of the betterment of gait in the posterior physical performance. Therefore, not including AO in the MI protocols (16, 17) may cause the control groups to not differ from the experimental groups reported. Similarly, to ensure the sharpness of MI in our protocol, we include an intra-session monitoring measure to verify that participants are achieving sufficiently effective imagery. Also related to the effectiveness of the vividness, the severity of the disease, and the appearance of cognitive impairment, may influence the superiority of physical therapy with MI. From the studies reviewed, two samples studied have a Montreal Cognitive Assessment score < 26 (14, 15), which implies cognitive impairment (63). On the other hand, when the studies evaluated cognition through the MiniMental State Examination, the participants could also present a wide range of cognitive performance with a normal score, even including a level of impairment consistent with dementia (64), due to the poor sensitivity for detecting cognitive impairment (including mild cognitive impairment) in PD, by comparing it to the Montreal Cognitive Assessment (65–67).

Apart from cognitive status, the assessment instruments used, and their ability to detect small changes, are relevant when analyzing the possible superiority of physical therapy interventions with MI compared to programs without MI. In this regard, scales and questionnaires, evaluated by a professional or self-reported by the participants, have a subjectivity bias and could be less sensitive to change (68), besides the ceiling effect that these tools have, which is accentuated in the evaluation of patients who have minimal or mild gait disturbance. From the previous results reported, Santiago et al. (15) evaluate the kinematics gait with a Motion Capture System, reporting a hip range improvement of 6.5%. The GAITimagery protocol includes the assessment with dynamometric platforms and inertial sensors that allow us to register spatiotemporal, dynamometric, and kinematics parameters from the gait performance. Likewise, we also incorporate common clinical measurement questionnaires and scales that will enable us to analyze the different percentages of improvement measured with both tools systems, psychometric and instrumental. In line with the measured variables, the primary outcome to estimate the sample size used in previous protocols of MI and physiotherapy of gait for people with PD, were the Hip range of motion (14, 69), the Visual analog scale of walking (16), the Functional independence measurement scale, and the UPDRS (35). On the other way, the primary outcome of the GAITimagery trial is gait speed, considered the most powerful biomarker of mobility (70), and is an objective parameter that does not depend on the evaluator, as clinical scales do. In this sense, the GAITimagery protocol will provide an objective and portraying parameter during PD course (26) for sample size estimation for future researchers.

Despite not reporting differences between groups, previous studies informed that gait physiotherapy with MI causes statistical changes in the total score of MiniBEST test, the Sensory orientation and Dynamic gait domain of balance assessment (14), the hip ROM, time of stance and swing phase of gait cycle (15), the mental section from UPDRS, and Schwab and England scale score for Activities of daily life (17). Furthermore, other authors have been informed of improvements in the Timed Up and Go test, the 10-meter walking test, and the score in the MiniBest balance test (20) when combining MI with dual-task; and MI combined with Neurofeedback has demonstrated improvement in endurance walking (23).

Even when we hypothesize that MI added to gait training could achieve a greater effect than detached gait training in people with PD, several factors or possible covariates must take into account to control the influence on the variance in dependent outcomes, such as the number of training sessions completed, average time elapsed between the medication doses intake and the start of the physiotherapy sessions, the stage of PD severity, and other between-groups factors like sex or level of activities in daily life, as the GAITimagery protocol contemplate. The methodological considerations that the GAITimagery trial has incorporated in the future development of this trial will allow us to analyze the effects of MI techniques in physiotherapy. The MI is an easily transferable to clinical practice technique because it does not require expensive materials or additional training for therapists, unlike other strategies such as neurofeedback or virtual reality. Nevertheless, it is necessary to study the ratio of gait improvement that can be explained by the MI aside from its superiority compared to not including MI in physiotherapy programs.

## Supplementary material

Supplementary material 1 Checklist SPIRIT.

Supplementary material 2 Committee approve.

Supplementary material 3 Informed consent form.
