# Supplementary material for: Gait physiotherapy with motor imagery in people with Parkinson’s disease: a protocol for randomized control GAITimagery trial
Source: Front Neurol. 2025 Jan 17;15:1508043. doi: 10.3389/fneur.2024.1508043 (PMC11783183; doi:10.3389/fneur.2024.1508043)
Supplement: Supplementary material 2 — Committee approve. [file Supplementary_file_2.pdf]

El comité Ético de Investigación en Humanos de la Comisión de Ética en Investigación Experimental de la Universitat de València,

CERTIFICA:

Que el Comité d'Ètica d'Investigació en Humans , en la reunión celebrada el día , una vez estudiado el proyecto de tesis doctoral : *"EFECTOS DE LA REHABILITACIÓN DE LA MARCHA CON IMAGINERÍA MOTORA EN PERSONAS CON ENFERMEDAD DE PARKINSON "*, con número de registro1557673 .

Cuyo/a responsable es D/Dña.

MARIA LUZ SANCHEZ SANCHEZ , dirigida por D/Dña. MARIA LUZ SANCHEZ SANCHEZ

ha acordado informar favorablemente el mismo.

Y para que conste, se firma el presente certificado

Av. Blasco Ibáñez, 13 tel: 963864109 vicerec.investigacio@uv.es
València 46010 fax: 963983221 www.uv.es/serinves

Firmado digitalmente por
PEDRO JESUS PEREZ ZAFRILLA
Cargo: Presidente del Comité de Ética de la Investigación en Humanos
Fecha: 11/02/2021 23:55:27 CET

The Ethics Committee of Research in Humans of the Ethics Commission in Experimental Research of University of Valencia,

CERTIFY:

Hereby certify that the Ethics Committee of Research in Humans, in the session which took place on , analysed the project of doctoral thesis entitled *"EFFECTS OF GAIT REHABILITATION WITH MOTOR IMAGERY IN PEOPLE WITH PARKINSON'S DISEASE "*, with register code 1557673 .

Whose researcher in charge is MARIA LUZ SANCHEZ SANCHEZ , and agreed with this project.

And in witness whereof, I hereby sign this certificate

Av. Blasco Ibáñez, 13 tel: 963864109 vicerec.investigacio@uv.es
València 46010 fax: 963983221 www.uv.es/serinves

Comité d'Ètica d'Investigació en Humans de la Comissió d'Ètica en Investigació Experimental de la Universitat de València,

CERTIFICA:

Que Comité d'Ètica d'Investigació en Humans , en la reunió que tingué lloc el dia , una vegada estudiat el projecte de tesi doctoral titulat:*"EFECTES DE LA REHABILITACIÓ DE LA MARXA AMB IMATGERIA MOTORA EN PERSONES AMB MALALTIA DE PARKINSON "*, amb codi de registre 1557673 .

que té com a responsable

MARIA LUZ SANCHEZ SANCHEZ , i que va dirigir MARIA LUZ SANCHEZ SANCHEZ ,

ha acordat emetre'n un informe favorablement .

I perquè així conste, signa aquest certificat.

Av. Blasco Ibáñez, 13 tel: 963864109 vicerec.investigacio@uv.es
València 46010 fax: 963983221 www.uv.es/serinves
