# Supplementary material for: Gait physiotherapy with motor imagery in people with Parkinson’s disease: a protocol for randomized control GAITimagery trial
Source: Front Neurol. 2025 Jan 17;15:1508043. doi: 10.3389/fneur.2024.1508043 (PMC11783183; doi:10.3389/fneur.2024.1508043)
Supplement: Supplementary material 3 — Informed consent form. [file Supplementary_file_3.docx]

DOCUMENTO DE CONSENTIMIENTO INFORMADO

Y COMPROMISO DE CONFIDENCIALIDAD

**1.- INFORMACIÓN AL SUJETO DE EXPERIMENTACIÓN**

*El proyecto de investigación para el cual le pedimos su participación se titula*:

“EFECTOS DE LA REHABILITACIÓN DE LA MARCHA CON IMAGINERÍA MOTORA EN PERSONAS CON ENFERMEDAD DE PARKINSON”. Puede leer más al respecto en el siguiente enlace:

<https://clinicaltrials.gov/study/NCT04788693>

Para que usted pueda participar en este estudio, es necesario contar con su consentimiento y que conozca la información básica necesaria para que dicho consentimiento, pueda considerarse verdaderamente informado. Lea detenidamente la siguiente información y si tiene alguna duda, exprésela antes de firmar este documento al investigador principal del proyecto personalmente, por teléfono o mediante correo electrónico. Los datos del investigador principal de este estudio aparecen en el presente documento.

La información básica que debe conocer es la siguiente:

a) *Objetivo del estudio*: Determinar el efecto de un programa de entrenamiento de marcha con imaginería motora en la marcha, la función motora y la percepción de la calidad de vida de personas con Enfermedad de Parkinson.

b) *Metodología a utilizar para el estudio, tipo de colaboración que se espera de usted y duración de dicha colaboración*:

- Se le solicita autorización para participar en un programa de entrenamiento fisioterápico para la enfermedad de Parkinson que pretende mejorar su forma de caminar, su función motora y su calidad de vida.
- Dicho programa se llevará a cabo en la Facultat de Fisioteràpia de la Universitat de València.
- Tendrá que realizar un total de 2 sesiones semanales de fisioterapia con una duración total de 60 minutos, en donde se practicarán diferentes ejercicios.
- Con el fin de determinar el efecto de las técnicas utilizadas, se requiere que usted participe en tres evaluaciones con una duración de aproximadamente 90 minutos cada una. Las evaluaciones se llevarán a cabo en el mismo centro de la intervención antes de que esta comience, al terminarla y a las cuatro semanas siguientes. En ellas usted debe contestar algunos cuestionarios acerca de su estado de salud y realizar pruebas de equilibrio y marcha de uso frecuente a nivel clínico. No se realizan pruebas invasivas.
- En la sesión número 6, se le realizará una evaluación de seguimiento en donde se observará su forma de andar, la cual dura aproximadamente 10 minutos.

c) *Procedimientos preventivos, diagnósticos y/o terapéuticos disponibles alternativos a los que se investigan con este estudio*: NO PROCEDE.

d) *Posibles molestias y* *riesgos de su participación en el estudio*: NO PROCEDE. Este estudio no supone ningún riesgo para su salud.

e) *Medidas para responder a los acontecimientos adversos*: NO PROCEDE

f) *Medidas para asegurar una compensación adecuada en el caso de daño*: NO PROCEDE

g) *Beneficios que se espera obtener con la investigación*: Las técnicas fisioterápicas empleadas podrían mejorar su función motora, marcha y calidad de vida. Sin embargo, existe la probabilidad que no experimente ningún cambio. Además, este estudio pretende mejorar el tratamiento fisioterápico de futuras personas con Enfermedad de Parkinson.

h) *Consecuencias de la no participación*: La participación en este estudio es voluntaria y puede cancelarse en cualquier momento. Si rechaza participar, no habrá consecuencias negativas para usted de ningún tipo.

i) *Posibilidad de retirada en cualquier momento y consecuencias*: Usted puede retirarse del proyecto en cualquier momento, sin tener que dar explicaciones, firmando la revocación del consentimiento que se incluye al final del documento. Su retirada no tendrá ninguna consecuencia negativa para usted, y será aceptada sin problemas por el equipo investigador. Si se retira del proyecto, puede decidir si los datos utilizados hasta ese momento deben borrarse o si se pueden seguir utilizando tras haberlos convertido en anónimos.

j) *¿Quién ha financiado el estudio?*: Sin financiación externa.

k) *¿Qué institución lo realiza?*: Universitat de València.

l) *Gratuidad por la participación*: Los y las participantes de la intervención no recibirán ningún tipo de compensación económica.

m) *Previsión de uso posterior de los resultados*: Los resultados del estudio solamente se utilizarán con fines de docencia, investigación y/o publicación científica de los resultados encontrados. Se respetará siempre la debida anonimización de los datos de carácter personal, de modo que los y las participantes de la investigación no resultarán identificados o identificables.

n) *Equipo investigador*: Profesoras M. Luz Sánchez y Constanza San Martín.

o) *Datos de contacto del investigador principal para aclaraciones o consultas*: Podrá solicitar cualquier explicación que desee sobre cualquier aspecto del estudio y sus implicaciones a lo largo del mismo contactando con:

- Prof. Constanza San Martín al correo electrónico Constanza.martin@uv.es
- Prof. M. Luz Sánchez al teléfono 96.398.38.55 (ext. 51268) o en el correo electrónico M.Luz.Sanchez@uv.es

p) El proyecto se realizará siguiendo los criterios éticos internacionales recogidos en la Declaración de Helsinki.

**2.- COMPROMISO DE CONFIDENCIALIDAD**

a) *Medidas para asegurar el respeto a la vida privada y a la confidencialidad de los datos personales*: Se han adoptado las medidas para garantizar la confidencialidad de los datos personales de los participantes del estudio, de acuerdo con la Ley De Protección de Datos de Carácter Personal (LOPD) 3/2018, de 5 de diciembre. **Todos sus datos e información médica que proporcione serán tratados con absoluta confidencialidad por los investigadores**. **Si los resultados del estudio fueran susceptibles de publicación en revistas científicas, en ningún momento se proporcionarán datos personales de las personas que han colaborado en esta investigación**.

b) *Medidas para acceder a la información relevante para usted que surjan de la investigación o de los resultados totales*: **Tiene derecho a acceder a la información generada sobre usted en el estudio, solicitándola al investigador principal**.

c) *Medidas tomadas por tratarse de un estudio anonimizado*: Se ha establecido un sistema de anonimización efectivo que no permite la identificación posterior del participante. Los consentimiento otorgados y los cuestionarios utilizados en el estudio se custodiarán de manera separada. **El uso de los resultados será con fines de docencia, investigación y/o publicación, y se respetará la anonimización de los datos personales**.

3.- **CONSENTIMIENTO** (EJEMPLAR PARA EL/LA PARTICIPANTE)

Don/Doña _________________________________________________________________________________________________,

mayor de edad, titular del DNI : _______________________, por el presente documento manifiesto que:

por el presente documento manifiesto que:

He sido informado/a de las características del Proyecto de Investigación titulado: “EFECTOS DE LA REHABILITACIÓN DE LA MARCHA CON IMAGINERÍA MOTORA EN PERSONAS CON ENFERMEDAD DE PARKINSON”.

He leído tanto el apartado 1 del presente documento titulado “información al sujeto de experimentación”, como el apartado 2 titulado “compromiso de confidencialidad”, y he podido formular las dudas que me han surgido al respecto. Considero que he entendido dicha información.

Estoy informado/a de la posibilidad de retirarme en cualquier momento del estudio.

En virtud de tales condiciones, consiento participar en este estudio.

Y en prueba de conformidad, firmo el presente documento en el lugar y fecha que se indican a continuación.

Valencia, ___________ de _____________________ de 20___.

| *Nombre y apellidos*  *del/ de la participante*:  Firma: | *Nombre y apellidos*  *del investigador principal*:  Firma: |
| --- | --- |

**REVOCACIÓN DEL CONSENTIMIENTO**

Revoco el consentimiento prestado en fecha __________________________ para participar en el proyecto titulado “EFECTOS DE LA REHABILITACIÓN DE LA MARCHA CON IMAGINERÍA MOTORA EN PERSONAS CON ENFERMEDAD DE PARKINSON” y, para que así conste, firmo la presente revocación.

En Valencia, a ________ de _________________________ de 20___.

| *Nombre y apellidos*  *del/ de la participante*:  Firma: | *Nombre y apellidos*  *del investigador principal*:  Firma: |
| --- | --- |

**3.- CONSENTIMIENTO** (EJEMPLAR PARA EL EQUIPO INVESTIGADOR)

Don/Doña _________________________________________________________________________________________________,

mayor de edad, titular del DNI : _______________________, por el presente documento manifiesto que:

por el presente documento manifiesto que:

He sido informado/a de las características del Proyecto de Investigación titulado: “EFECTOS DE LA REHABILITACIÓN DE LA MARCHA CON IMAGINERÍA MOTORA EN PERSONAS CON ENFERMEDAD DE PARKINSON”.

He leído tanto el apartado 1 del presente documento titulado “información al sujeto de experimentación”, como el apartado 2 titulado “compromiso de confidencialidad”, y he podido formular las dudas que me han surgido al respecto. Considero que he entendido dicha información.

Estoy informado/a de la posibilidad de retirarme en cualquier momento del estudio.

En virtud de tales condiciones, consiento participar en este estudio.

Y en prueba de conformidad, firmo el presente documento en el lugar y fecha que se indican a continuación.

Valencia, ___________ de _____________________ de 20___.

| *Nombre y apellidos*  *del/ de la participante*:  Firma: | *Nombre y apellidos*  *del investigador principal*:  Firma: |
| --- | --- |

**REVOCACIÓN DEL CONSENTIMIENTO**

Revoco el consentimiento prestado en fecha __________________________ para participar en el proyecto titulado “EFECTOS DE LA REHABILITACIÓN DE LA MARCHA CON IMAGINERÍA MOTORA EN PERSONAS CON ENFERMEDAD DE PARKINSON” y, para que así conste, firmo la presente revocación.

En Valencia, a ________ de _________________________ de 20___.

| *Nombre y apellidos*  *del/ de la participante*:  Firma: | *Nombre y apellidos*  *del investigador principal*:  Firma: |
| --- | --- |
